# Wenn Notarzt und Telenotarzt gemeinsam Leben retten: Eine Fallbeschreibung für die Anwendung der prähospitalen Telemedizin bei einer kreislaufinstabilen ventrikulären Tachykardie

**DOI:** 10.1007/s00101-020-00872-w

**Published:** 2021-01-15

**Authors:** A. Follmann, H. Schröder, G. Neff, R. Rossaint, F. Hirsch, M. Felzen

**Affiliations:** 1 Klinik für Anästhesiologie, Uniklinik RWTH Aachen, Pauwelsstraße 30, 52074 Aachen, Deutschland; 2 umlaut telehealthcare GmbH, Aachen, Deutschland; 3 Rettungsdienst Kreis Euskirchen, Euskirchen, Deutschland

**Keywords:** Notfallmedizin, Telemedizin, Ventrikuläre Tachykardie, Zweitmeinung, Supervision, Emergency medicine, Telemedicine, Ventricular tachycardia, Second opinion, Supervision

## Abstract

Die Telemedizin ist in einigen Regionen Deutschlands bereits fester Bestandteil des Rettungsdienstes. Dieser Fallbericht handelt von einem Telenotarzteinsatz, bei dem die initiale Telekonsultation durch eine an der Einsatzstelle befindliche Notärztin erfolgte. Der Patient hatte eine lebensbedrohliche ventrikuläre Tachykardie mit zunehmender Kreislaufinstabilität. Dies stellte für die noch unerfahrene Notärztin eine extrem herausfordernde Einsatzsituation dar. Sie entschied sich, telemedizinische Unterstützung in Anspruch zu nehmen. So konnte sie angeleitet werden, bei dem instabilen Patienten einen i.o.-Zugang, eine medikamentöse Therapie und eine elektrische Kardioversion durchzuführen. Die Zusammenarbeit mit dem Telenotarzt ermöglichte der noch unerfahrenen Notärztin, eine leitlinienkonforme Therapie durchzuführen sowie den Patienten zeitnah stabilisiert ins Krankenhaus zu transportieren.

## Abstract (en)

Telemedicine has already entered the rescue service in some regions of Germany. This case description is about a telemedical emergency physician case where an emergency doctor was also at the scene of the emergency. The patient had a life-threatening ventricular tachycardia and became hemodynamically unstable. The emergency physician was still inexperienced and overwhelmed by the complex situation. She decided to contact the tele-emergency medical services (tele-ems) and could then be instructed to perform intraosseous access, drug treatment and electrical cardioversion in the unstable patient. The cooperation with the tele-ems physician enabled the still inexperienced emergency physician to perform a guideline-compliant treatment and to transport the stabilized patient to the hospital in a timely manner

Die Telemedizin nimmt im deutschen prähospitalen Rettungsdienst immer weiter an Bedeutung zu. Bereits seit 2014 kommt in Aachen ein kassenfinanziertes holistisches Telemedizinsystem unter Trägerschaft der Berufsfeuerwehr zur Anwendung, das neben der reinen Liveübertragung von Vitaldaten, Fotos und Video auch eine leitlinienkonforme und algorithmenbasierte Diagnose- und Behandlungssoftware beinhaltet [1, 6, 7, 10]. Die Telemedizin gewährleistet die Verfügbarkeit ärztlicher Kompetenz im Rettungsdienst trotz weiter Distanz [2–5]. Bei komplexen Einsatzlagen bietet sie Unterstützung für die Rettungskräfte vor Ort, sowohl medizinisch als auch organisatorisch. Wenn kein Notarzt vor Ort ist, bietet der Telenotarzt zudem eine Rechtssicherheit für die anwesenden Rettungsassistenten oder Notfallsanitäter, da er die Gesamtverantwortung für die getroffenen medizinischen Entscheidungen trägt [10]. Der erfolgreiche Einsatz in zahlreichen Modellregionen hat dazu geführt, dass mittlerweile Bundesländer wie Bayern und Nordrhein-Westfalen eine flächendeckende Einführung eines solchen Systems in Aussicht stellen. Neben den zahlreich beschriebenen Vorteilen gibt es aber auch Kritik. Notärzte sehen teilweise ihre Zukunft gefährdet und befürchten, dass sie durch einen telemedizinischen Kollegen ersetzt werden könnten. Das folgende Fallbeispiel soll zeigen, warum ein Nebeneinander beider ärztlicher Versorgungseinheiten im Rettungsdienst in Deutschland sinnvoll ist und dazu beitragen kann, die Qualität der Patientenversorgung zu steigern und suffizient Leben zu retten.

## Falldarstellung

### Anamnese

Im Frühjahr 2017 wurden abends der Rettungswagen und das Notarzteinsatzfahrzeug einer ländlichen Region von der Rettungsleitstelle zu einem „unklaren Notfall“ alarmiert.

Der männliche Patient war 54 Jahre alt und hatte keine relevanten Vorerkrankungen angegeben. Er litt unter Adipositas und hatte eine familiäre Prädisposition für kardiovaskuläre Ischämien. Nikotinabusus war ein weiterer vorliegender Risikofaktor. Eine medikamentöse Dauertherapie bestand nicht. Allergien wurden verneint.

Er klagte über plötzlich aufgetretenen, starken Schwindel und Übelkeit. Diese Symptome waren kurz vor Absetzen des Notrufes plötzlich aufgetreten und seitdem anhaltend. Nach einer initialen Erstversorgung durch die Notärztin vor Ort konsultierte diese einen Telenotarzt zum gleichen Fall. Dabei wurde der Patient als wach, ansprechbar (GCS 15) und kaltschweißig beschrieben. Eine medikamentöse Therapie war bereits mit 5 mg Metoprolol i.v. bei bestehender Tachykardie zur Frequenzlimitation begonnen worden, zeigte aber keinerlei Therapieerfolge. Der peripher venöse Zugang war aufgrund der bestehenden Kaltschweißigkeit akzidentiell verloren gegangen. Die Notärztin hatte den Patienten bereits in einer kardiologischen Fachklinik angemeldet, sich dann aber aufgrund der langen Fahrtzeit von ca. 40 min entschieden, den Telenotarzt zusätzlich zur Einsatzunterstützung zu konsultieren.

### Klinischer Befund

Zu Beginn der Telekonsultation zeigte sich im übertragenen Echtzeit-EKG eine Tachykardie mit breiten Kammerkomplexen und einer Herzfrequenz über 200/min. Periphere Pulse waren laut Notärztin vor Ort nicht tastbar. Dennoch konnte in der Pulsoxymetrie eine periphere Sauerstoffsättigung von 92 % gemessen werden. Weiterhin war kein zusätzliches A‑, B‑, D‑ oder E‑Problem festgestellt worden, sodass der Fokus zunächst auf das kardiologische Problem gelegt wurde.

### Diagnose

Zusammenfassend zeigte sich für den Telenotarzt ein kreislaufinstabiler Patient mit einer anhaltenden ventrikulären Tachykardie (VT), der noch wach und ansprechbar war.

Der Telenotarzt konnte dem Team vor Ort verdeutlichen, dass es sich bei dem vorliegenden Fall um einen lebensbedrohlichen Zustand handelte und ein zügiger Transport in eine kardiologische Fachklinik ohne weitere Therapie vor Ort keine ausreichende Option darstellte.

### Therapie und Verlauf

Bei schlechter peripherer Füllung und mäßigem Venenstatus war es nicht möglich, innerhalb eines adäquaten Zeitintervalls einen neuen peripheren Venenzugang zu etablieren. Daher wurde die Notärztin vor Ort durch den Telenotarzt erfolgreich angeleitet, einen i.o.-Zugang an der proximalen Tibia zu etablieren. Da der Patient zu diesem Zeitpunkt bei fortschreitender Instabilität nur noch bedingt ansprechbar war, wurde auf eine lokale Betäubung verzichtet. Der Telenotarzt wies währenddessen die Besatzung des Rettungswagens an, eine Kurzinfusion (5 %ige Glucose-Lösung) mit 300 mg Amiodaron vorzubereiten, um alle möglichen Therapiemaßnahmen im Falle eines Misslingens der Kardioversion auszuschöpfen.

## Präklinischer Verlauf

**Figure 1:**
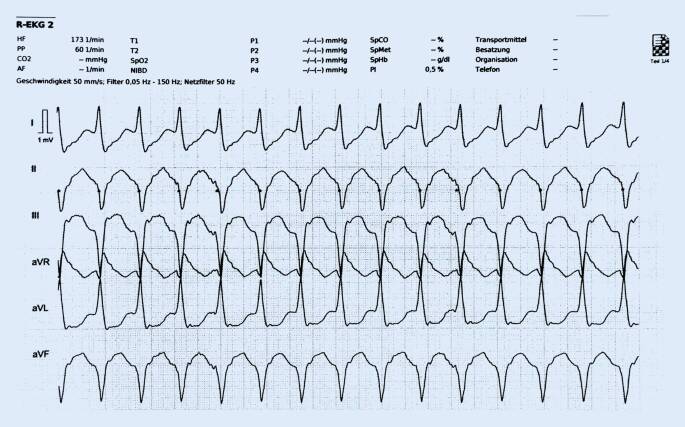

Bei weiter anhaltender VT (Abb. 1) war der Patient nur noch auf Schmerzreiz erweckbar. Der Telenotarzt wies die Besatzung des Rettungswagens an, zügig die Klebeelektroden des Defibrillators für eine elektrische Kardioversion vorzubereiten und am Patienten anzubringen. Die Notärztin vor Ort entschied sich in Absprache mit dem Telenotarzt für eine Kurznarkose mit Midazolam und Fentanyl. Unter telemedizinischer Anleitung gelang es der Notärztin, den Patienten mit einer synchronisierten elektrischen Kardioversion (150 J biphasisch) zu therapieren: Im Anschluss zeigte sich ein Sinusrhythmus mit normfrequenter Überleitung und suffizientem Auswurf bei wieder tastbarem Puls. Der Blutdruck wurde nichtinvasiv mit 115/78 mm Hg gemessen.

**Figure 2:**
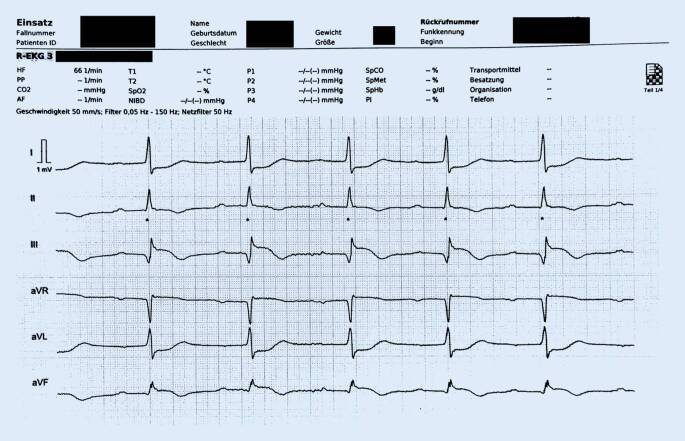

Auf dem Monitor des Telenotarztes war bereits in der Ableitung III eine Hebung der ST-Strecke im EKG zu sehen. Daher forderte er die Kollegin vor Ort auf, ein 12-Kanal-EKG anzufertigen. Dieses wurde telemedizinisch übertragen (Abb. 2). Hier zeigte sich das Bild eines ST-Hebungs-Infarktes (STEMI) der Hinterwand. Die Notärztin vor Ort wurde unverzüglich vom Telenotarzt über diese Diagnose informiert. Die leitliniengerechte Therapie des Hinterwandinfarkts wurde gemeinsam besprochen und anschließend von der Notärztin vor Ort durchgeführt. Der Telenotarzt kontaktierte den diensthabenden Kardiologen in der bereits vorab erwähnten Fachklinik und konnte ihm das EKG per Fax, ebenfalls vorab, zusenden. Der Patientenzustand sowie präklinische Diagnostik und Therapie wurden mit dem ärztlichen Kollegen im aufnehmenden Krankenhaus ausführlich besprochen und ein zügiger Transport in die Zielklinik angestrebt. Dieser erforderte knapp 40 min. Zusätzlich zum Monitoring im Rettungswagen unter Aufsicht der anwesenden Notärztin erfolgte auch eine telemedizinische Transportbegleitung.

Während des Transports war der Zustand des Patienten stabil. Bei fortbestehendem Sinusrhythmus zeigte sich eine stabile Hämodynamik mit nichtinvasiv gemessenen Blutdruckwerten von durchgehend ca. 120/80 mm Hg als Zeichen des suffizienten, kardialen Auswurfs. Die Sauerstoffsättigung blieb unter Sauerstoffinsufflation über die Reservoir-Maske stabil.

## Klinischer Verlauf

Das Herzkatheter-Team wurde von der Zielklinik vorab alarmiert, sodass eine Akutintervention umgehend bei Eintreffen des Patienten erfolgen konnte.

In der invasiven Diagnostik zeigten sich eine ausgeprägte dilatative Koronaropathie sowie ein älterer Verschluss der rechten Koronararterie (RCA). Ein Rekanalisierungsversuch blieb frustran. Der Patient konnte am selben Tag kreislaufstabil und beschwerdefrei auf die kardiologische Intensivstation übernommen werden. Im Verlauf kam es jedoch erneut zu einer anhaltenden VT, die bei Kreislaufstillstand eine Reanimation mit Defibrillation erforderte. Nach nahezu unmittelbarem ROSC kam es zu rezidivierenden VT und erneuter Kardioversion. Unter Amiodaron- und Lidocainaufsättigung wurden vorerst keine weiteren VT verzeichnet, jedoch traten diese nach Absetzen der Medikation erneut auf. Für den Patienten wurde schließlich die Implantation eines automatischen implantierbaren Kardioverter-Defibrillator (AICD) geplant.

## Diskussion

Die erfolgreiche Anwendung der Telemedizin ist in vielen Studien nachgewiesen worden [11]. Daraus resultierend wird in immer mehr Kommunen ein Telenotarztsystem installiert, das parallel zum Notarztwesen im rettungsdienstlichen Routinebetrieb tätig ist. Aachen hält als erste Stadt Deutschlands seit 2014 einen Telenotarzt im 24-h-Betrieb als strukturelle Ergänzung zum konventionellen bodengebundenen Notarztsystem vor [8]. Mit knapp 15.000 Einsätzen seit Einführung 2014 konnte die Notarztquote bei den Rettungsdiensteinsätzen der Stadt Aachen (Stand 2018) von 36 auf 20,5 % gesenkt werden. Dadurch haben jedoch nicht weniger Patienten einen Arztkontakt im Rahmen der prähospitalen Versorgung.

Die Telemedizin bietet eine zukunftsfähige Alternative und kompetente Ergänzung der Notfallmedizin, besonders in Kreisen des ländlichen Raums. Dabei folgen die Anforderungen an den Telenotarzt in Aachen den Strukturempfehlungen der DGAI [9]: Er verfügt mindestens über einen Facharztstandard in der Anästhesiologie und die Zusatzbezeichnung Notfallmedizin, hat an einem zertifizierten Reanimations- und Traumakurs teilgenommen und mindestens 500 konventionelle Notarzteinsätze absolviert. Eine zusätzliche Schulung über einen Zeitraum von 4 Tagen beinhaltet eine Einweisung in die Technik sowie Kommunikationstrainings.

Neben vielen weiteren Einsatzindikationen (Schmerztherapie bei traumatisch bedingten Einfachverletzungen, Hypertension, Schlaganfall, Herzrhythmusstörungen, Hypoglykämie etc.) ist auch das akute Koronarsyndrom ein Krankheitsbild, was telemedizinisch unterstützt werden kann und gerade bezüglich der Leitlinientreue und Versorgungsqualität des Patienten einer Therapie durch den Notarzt vor Ort mindestens gleichwertig ist [2]. Durch das emotionsfreie Abarbeiten von Algorithmen sowie einen breiten Datenbankzugriff können Versorgungen standardisiert erfolgen und organisatorische Aufgaben, wie die Voranmeldung, inkl. Befundübertragung, in der aufnehmenden Klinik früh eingeleitet werden, ohne personelle Ressourcen der Notfallversorgung vor Ort zu verbrauchen.

Der Telenotarzt soll dabei immer als Ergänzung zum bestehenden Notarztsystem und als weiteres Strukturelement des Rettungsdienstes verstanden werden. Einen Ersatz für den Notarzt stellt er nicht dar. Häufig wird aber die Telemedizin kritisiert, genau darauf abzuzielen. Das beschriebene Fallbeispiel macht jedoch deutlich, dass ein Zusammenspiel beider Versorgungsstrategien sinnvoll ist. Die Notärztin vor Ort absolvierte während des beschrieben Falls ihre zweite Schicht auf einem Notarzteinsatzfahrzeug. Trotz guter Ausbildung und Erlangen der Zusatzbezeichnung Notfallmedizin war sie aufgrund der komplexen Einsatzsituation in ihrer Handlungsweise unsicher. Wäre eine vergleichbare Situation in der Klinik aufgetreten, so wäre es selbstverständlich sowohl zum Wohle des Patienten als auch aus Eigenschutzaspekten, dass man einen Oberarzt involviert. Der Telenotarzt bietet nunmehr die Möglichkeit, genau dies auch prähospital zu tun.

In diesem Sinne bietet der Telenotarzt nicht nur Notfallsanitätern die Möglichkeit, ein rechtssicheres Handeln entsprechend geltender Leitlinien mit Delegation von Medikation zu ermöglichen, sondern selbst erfahrenen Notärzten eine zeiteffiziente Arbeit vor Ort durch die Übernahme organisatorischer Aufgaben. Doch gerade bei Berufsanfängern aller Berufsgruppen kann eine mögliche Handlungsangst durch das kollegiale Ersuchen einer Zweitmeinung sowohl durch Notfallsanitäter als auch durch ärztliches Rettungsdienstpersonal vermieden werden und ermöglicht eine optimale Patientenversorgung. Sicherlich ist die Unerfahrenheit der hier genannten Notärztin ein menschliches Hindernis zur schnellen Therapieentscheidung dieses Patientenfalls. Doch gerade in dieser Situation führt die zügige Einschließung eines Telenotarztes zu einer entsprechenden Kompensation des Zeitverlustes.

Die ventrikuläre Tachykardie im beschriebenen Fall war initial nicht pulslos, sondern stellte einen typischen Periarrestrhythmus dar. Bei fortbestehender Rhythmusstörung konnte keine ausreichende Auswurfleistung mehr durch das Herz erbracht werden; eine zunehmende Vigilanzminderung stellte sich ein.

Die Notärztin erkannte die Tachykardie und begann eine medikamentöse Therapie mit Metoprolol. Die β‑Blockade war jedoch bezüglich der Frequenzlimitation frustran sowie entsprechend dem ERC-Algorithmus für tachykarde Herzrhythmusstörungen nicht das Medikament der Wahl. Weiterhin kann Metoprolol aufgrund seiner negativ-inotropen Wirkung die Hämodynamik in dieser Situation noch zusätzlich verschlechtern. Rückwirkend betrachtet, führte die Ischämie des Myokards zu der malignen Rhythmusstörung, und mit abnehmender Herzauswurfleistung wurde der Patient hämodynamisch instabil. Ein typisches Zeichen war die kalte, nasse Haut. Diese erschwerte die Fixierung des bereits etablierten i.v.-Zugangs, was einen akzidentiellen Verlust zur Folge hatte. Die Notärztin hatte in dieser Situation nicht das Medikament der Wahl eingesetzt, aber die hämodynamische Instabilität und dessen Lebensbedrohlichkeit für den Patienten richtig eingestuft und daher die korrekte Zuweisung in eine kardiologische Fachklinik initiiert. Doch vor einem knapp 40-minütigen Transport entschied sie sich, zusätzlich die Meinung des Telenotarztes einzuholen, der an den entsprechenden Rettungswagen angebunden war.

Zwar stehen einem Telenotarzt manche Informationen von der Einsatzstelle nicht zur Verfügung, wie beispielsweise sensorische Eindrücke oder ein kontinuierlicher „blickdiagnostischer“ Eindruck des Patienten. Aber gerade dadurch wird der Blick auf das Wesentliche gelenkt. So konnte aufgrund des telemetrisch übertragenen EKG zügig die Diagnose einer ventrikulären Tachykardie gestellt werden, die mit der Notärztin vor Ort und dem Team kommuniziert wurde. Da ein periphervenöser Zugang nicht zeitnah zu etablieren, aber dringend erforderlich war, empfahl der Telenotarzt der Kollegin vor Ort, einen i.o.-Zugang zu legen. Hierbei konnte er die Notärztin bei ihrer ersten Anwendung des i.o.-Zugangs unterstützend anleiten. Bei zunehmender Kreislaufinstabilität wurde schließlich die synchrone elektrische Kardioversion durchgeführt, die eine Konversion in einen Sinusrhythmus bewirkte. Auch hier konnte sich der Telenotarzt weiter auf die Vitaldaten des Patienten konzentrieren und in der Echtzeit-Kurve des EKG den Verdacht auf eine ST-Hebung in der Ableitung III stellen, der sich schließlich im 12-Kanal-EKG bestätigte. Es erfolgte die leitliniengerechte Therapie bei bestehendem STEMI. Die Kreislaufsituation stabilisierte sich; ein peripherer Puls war wieder palpabel.

Ein weiterer Vorteil der Telemedizin ist in der technisch-organisatorischen Innovation zu sehen. So konnte der involvierte Telenotarzt Dokumente wie beispielsweise das Einsatzprotokoll, die EKG, aber auch Fotos von alten Arztbriefen an die aufnehmende Fachklinik weiterleiten. Der Fall konnte mit dem dortig diensthabenden Kollegen besprochen und ausführlich diskutiert werden, lange bevor der Patient dort eintraf. So wurde die zielgerichtete Therapie der interventionellen Koronarangiographie beschleunigt und der Patient schließlich mit mehrfachen Stent-Implantaten definitiv versorgt.

Hätte die Notärztin vor Ort nicht den Telenotarzt kontaktiert, hätte sie sich evtl. für einen zügigen Transport in die angestrebte Zielklinik entschieden. In diesem Fall wäre aufgrund der Transportstrecke ein Kreislaufstillstand hochwahrscheinlich gewesen, der dann eine sofortige Defibrillation der VT und den Beginn von Reanimationsmaßnahmen erfordert hätte. Doch dadurch, dass die Kollegin vor Ort sich eine Zweitmeinung einholen und den Telenotarzt konsultieren konnte, wurde der Patient zielgerichtet einer Therapie zugeführt, die ihm letztendlich das Leben retten konnte. Dies war im Beschriebenen nur durch den gemeinsamen Einsatz der Notärztin vor Ort und des Telenotarztes aus der Ferne möglich.

Die Forderung nach einer hohen notärztlichen Qualifikation schließt sicherlich auch eine hochwertige und zeitgemäße Einarbeitung junger Kolleginnen und Kollegen ein, wenn man eine ausreichende Zahl an ärztlichen Fachkräften langfristig gewährleisten möchte [12]. Daraus resultierend ergibt sich auch die Möglichkeit einer kollegialen telemedizinischen Supervision von jungen Notärzten sowie die Option einer telemedizinischen Unterstützung auch für erfahrene Kollegen vor Ort. Dieser Fall ist nur einer von vielen, zeigt aber bereits die Vielfältigkeit und das große Nutzspektrum einer telemedizinischen Unterstützung in der Notfallmedizin. Die deutschlandweite Entwicklung der Telemedizin kann dabei zu einer kompetenten medizinischen Versorgung beitragen.

Zusätzlich ist dies ein schönes Beispiel für zukunftsweisende Fehlerkultur in der Medizin. Häufig hindert die Sorge vor Schuldzuweisung und Versagen junge Kollegen daran, Fehler, mangelndes Wissen oder Unsicherheit in einer kritischen Situation einzugestehen. Die notärztliche Kollegin hat ihre Grenzen jedoch erkannt und verfügbare Hilfe in Anspruch genommen. Ganz im Sinne von modernen Konzepten der Patientensicherheit und des Crew Resource Management hat dies zum bestmöglichen Outcome des Patienten geführt.

## Fazit für die Praxis

- Telemedizin ermöglicht eine präklinische Supervision unerfahrener, aber auch erfahrener Notärzte.
- Ein Telenotarzt kann dem Notarzt vor Ort eine effiziente Unterstützung und Ergänzung bieten.
- Eine offene Fehlerkultur erhöht die Patientensicherheit.
